# Fulminant Epstein-Barr virus - infectious mononucleosis in an adult with liver failure, splenic rupture, and spontaneous esophageal bleeding with ensuing esophageal necrosis: a case report

**DOI:** 10.1186/1752-1947-8-35

**Published:** 2014-02-05

**Authors:** Daniel Busch, Sarah Hilswicht, Dominik S Schöb, Klaus T von Trotha, Karsten Junge, Nikolaus Gassler, Son Truong, Ulf P Neumann, Marcel Binnebösel

**Affiliations:** 1 Department of General, Visceral and Transplantation Surgery, RWTH Aachen University Hospital, Pauwelsstrasse 30, 52074 Aachen, Germany; 2 Institute of Pathology, RWTH Aachen University Hospital, Pauwelsstrasse 30, 52074 Aachen, Germany

**Keywords:** Infectious mononucleosis, Epstein-Barr virus, Esophageal necrosis, Hepatic failure

## Abstract

**Introduction:**

Infectious mononucleosis is a clinical syndrome most commonly associated with primary Epstein-Barr virus infection. The majority of patients with infectious mononucleosis recovers without apparent sequelae. However, infectious mononucleosis may be associated with several acute complications. In this report we present a rare case of esophageal rupture that has never been described in the literature before.

**Case presentation:**

We present the case of an 18-year-old Caucasian man affected by severe infectious mononucleosis complicated by fulminant hepatic failure, splenic rupture and esophageal necrosis.

**Conclusions:**

Although primary Epstein-Barr virus infection is rarely fatal, fulminant infection may occur - in this case leading to hepatic failure, splenic rupture and esophageal necrosis, subsequently making several surgical interventions necessary. We show here that infectious mononucleosis is not only a strictly medical condition, but can also lead to severe surgical complications.

## Introduction

Infectious mononucleosis (IM) is most often associated with Epstein-Barr virus (EBV), one of the most common human viruses, and a member of the family of human herpes virus [1]. IM is also called ‘kissing disease’ since infection with EBV is transmitted mostly via saliva. When infected during childhood, EBV usually causes only mild symptoms resulting in a lifelong dormant viral infection [1]. However, if primary infection occurs during adolescence, EBV leads to IM in 35% to 50% of the cases [2]. Typically, IM leads to the characteristic triad of fever, pharyngitis and lymphadenopathy (cervical, axillary and inguinal) that might last for up to several months and can be accompanied by splenomegaly. In the rare case of a severe infection, IM may cause hematologic or neurological complications, splenic rupture, upper airway obstruction, acute myocarditis, as well as mucosal edema and leads to increased bleeding tendency [2]. Although primary EBV infection is rarely fatal, fulminant infection may occur: fatal fulminant hepatitis and fulminant hepatic failure have been described [3].

We present the uncommon occurrence of a fulminant hepatic failure, splenic rupture and esophageal necrosis complicating a primary EBV infection in an 18 year-old Caucasian man.

## Case presentation

An 18-year-old Caucasian man was admitted to a peripheral hospital, presenting with a seven-day history of fever and a deteriorating general condition. An initial physical examination showed swollen cervical, submandibular and inguinal lymph nodes, enlarged tonsils and a significant elevation in serum liver enzyme levels. During the course of hospitalization, he showed a further increase of serum liver enzymes accompanied by distinct thrombocytopenia. On the sixth day after hospitalization, intermittent diffuse, mucosal bleeding with intermittent hemoptysis and tarry stools occurred. Laboratory testing showed an impaired coagulation due to deteriorated liver synthesis. Because of his persistent anemia despite transfusion of four red cell concentrates, a computed tomography (CT) scan of his thorax and abdomen was performed, showing pleural effusion, ascites, lymphadenopathy with enlarged cecal and portal lymph nodes up to 2.2cm, as well as hepatosplenomegaly (spleen 21cm and liver 18cm, transversal measurement respectively).

The next day, mucosal bleeding continued and the condition of the patient worsened. He was subsequently transferred to the intensive care unit (ICU) of our hospital. Laboratory parameters on admission showed severely impaired coagulation (29% prothrombin time (PT), fibrinogen not detectable (<0.3g/L), 44% antithrombin III (AT III), platelets 33G/L) and liver dysfunction (bilirubin 8.2mg/dL, direct bilirubin 5.2mg/dL, albumin 24g/L and plasma cholinesterase (PCHE) 1540U/L).

Our patient presented with diffuse bleeding of the oropharynx mucosa, which was treated with packing. Bleeding of the tracheal mucosa caused a significant impairment of his respiratory situation. Thus protective intubation of our patient followed. Bronchoscopy was performed and blood clots were removed. Because of his hemodynamic instability, blood transfusion was required.

However, after this episode his hemoglobin levels stabilized. A pulmonary focus after diffuse pulmonary bleeding was suspected to be the origin of the newly developing fever (>38.6°C) and he was subsequently treated with levofloxacin. One day after the initiation of antibiotic therapy our patient developed generalized exanthema, which quickly spread, progressing in intensity and turning darker with a more livid discoloration in the head region over the next three days. Levofloxacin was stopped and antibiotic therapy was switched to ceftazidime. Suspecting a specific viral infection, further analyses were performed. Virology was negative for hepatitis A, B, C virus (HAV, HBV, HCV), human immune deficiency virus (HIV) and cytomegalovirus (CMV), but positive for EBV infection (1.8 × 7× 10^5^ replications measured by polymerase chain reaction (PCR)). Because of the fulminate course, bacterial superinfection was suspected. Extensive microbiological screening was performed, but no specific bacterial or fungal germ could be detected. Our patient developed increasing amounts of ascites, and pleural effusions as well as sepsis with a multiorgan dysfunction syndrome (MODS) resulting in liver and kidney failure. Due to anuria, hemofiltration was required at this point.

**Figure 1:**
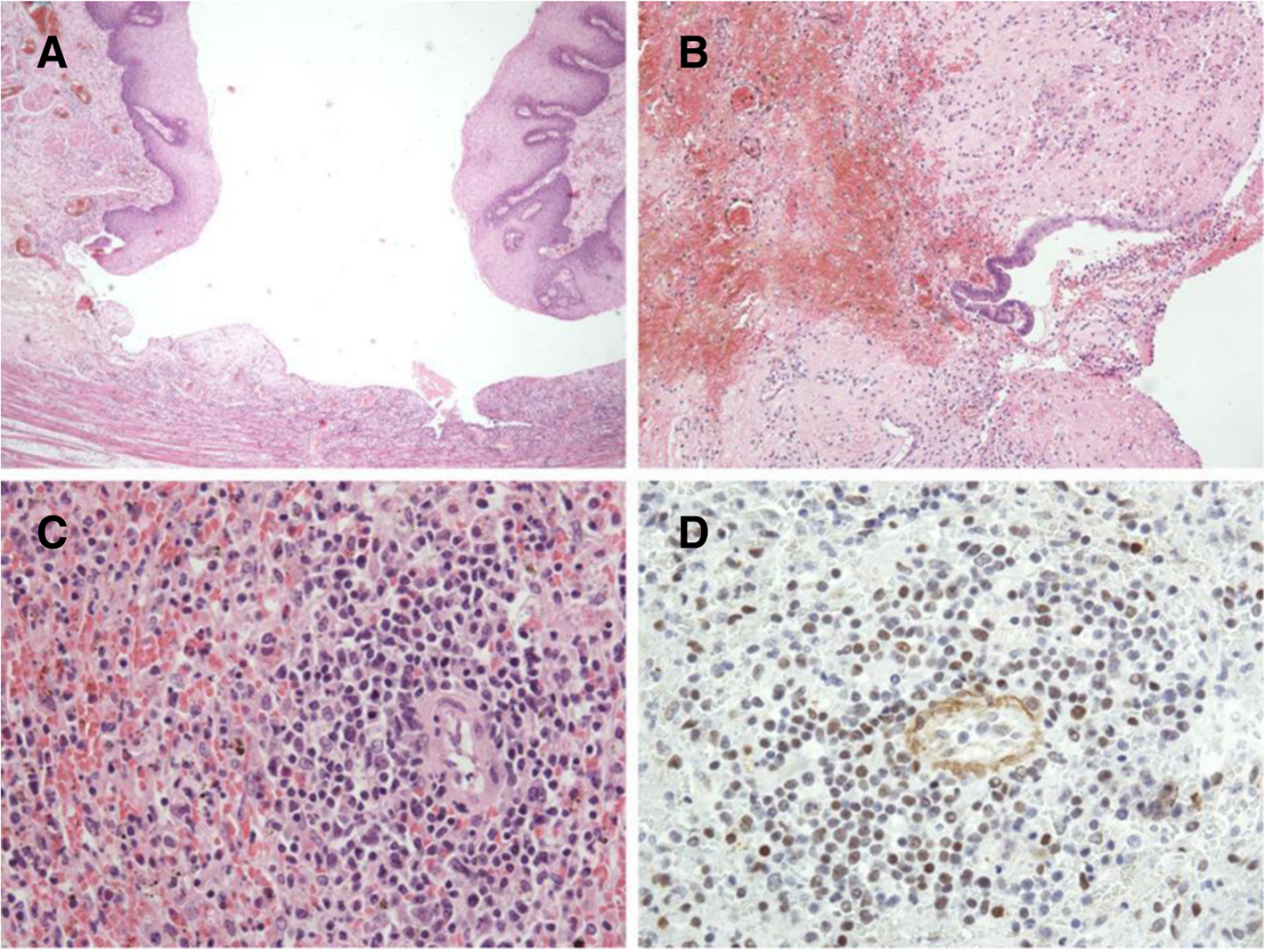
**Histological sections from several affected organs. (A)** Duodenal and **(B)** gastric mucosa are injured from florid inflammation with bleeding. **(C)** White pulpa of the splenic parenchyma is diminished and paralleled by hyperemia. **(D)** In the splenic parenchyma some lymphocytes immunostained against Epstein-Barr virus, latent membrane protein (clone CS.1-4; Dako, Hamburg, Germany) are positive.

On the morning of the 21^st^ day after hospitalization severe oral hemorrhage, with a significant drop in his hemoglobin level, occurred. Hemorrhagic shock developed rapidly. The gastric tube showed bloody contents so diagnostic esophagogastroduodenoscopy was performed. Endoscopy revealed significant arterial bleeding from a wide ulcer located in the distal esophagus. The bleeding could not be stopped endoscopically due to the intensity of the bleeding. An abdominal ultrasound scan showed moderate amounts of free fluid around the spleen as well as a central splenic lesion. Suspecting a spontaneous splenic rupture, our patient was transferred to the operating theater immediately. After median laparotomy, the intraoperative exploration showed massive enlargement of his spleen and liver. There were two subcapsular lacerations (Moore grade II) of the liver with a gentle venous bleeding in segment II/III and IVb, which were successfully treated by thermocoagulation. His spleen showed a grade IV rupture with involvement of the splenic hilus. Due to hemodynamic instability and general hemorrhagic diathesis, a splenectomy was performed. Further exploration of the abdomen revealed a covered perforation of a gastric ulcer in the region of the cardia, which was closed with a full-thickness running suture using 4/0 polydioxanone. Under intraoperative esophagogastroscopy a second bleeding ulcer was reliably identified in the esophagus and closed by transhiatal full-thickness suture using 4/0 polydioxanone. Ventral *Dor*-hemifundoplication was performed to adequately support the esophageal integrity. A left-sided chest tube was inserted to treat pleural effusion, and a temporary abdominal closure with an absorbable mesh (vicro) was performed. After surgery and massive transfusion with a total of 34 red blood cell concentrates, 40 packs of fresh frozen plasma, six thrombocyte concentrates and 1000 IU prothrombin complex (PPSB), our patient was transferred back to the ICU in a hemodynamically stable condition with only moderate catecholamine support. Histopathological findings of tissue samples collected during the surgical intervention showed gastric mucosal injury from florid inflammation with bleeding as given in Figure 1. The splenic parenchyma marked positive against EBV, also shown in Figure 1.

On the fifth postoperative day, there was a transition of drainage quality and our patient developed recurrent fever as well as rapidly increasing signs of infection. An esophagogastroduodenoscopy was performed. The endoscopy revealed a full-thickness necrosis of the distal esophagus from 30 to 38cm. Since there was no chance of an endoscopic or surgical restitution of the continuity of the esophagus, a discontinuity resection of the esophagus with proximal gastrectomy, collar salivary fistula and terminal gastrostomy was performed.

The following postoperative course was prolonged due to a very slow regeneration of liver and renal function as well as persistent respiratory insufficiency due to recurrent pneumonia. Hemofiltration was stopped the 25^th^ day after surgery when diuresis was gradually restored. On the 41^st^ postoperative day our patient started spontaneous breathing and artificial ventilation could be stopped. Our patient gradually recovered and was transferred to a standard care unit on the 50^th^ postoperative day. The further clinical course was uneventful. Our patient slowly recovered and could be discharged on the 86^th^ postoperative day with an epithelialized laparostoma, an enteral discontinuity with a collar salivary fistula and complete nourishment via a feeding tube inserted through the gastrostomy.

Hospital readmission was necessary six months following the initial surgery because of cachexia (body mass index 13kg/m^2^) due to malnutrition requiring an adjustment of the nutritional protocol. At this time, EBV infection was still present with a viral load of 4×7×10^3^ GE/mL. Enteral nutrition was improved and our patient gradually gained weight and strength.

One year after the discontinuity resection of the esophagus, the reconstruction of the intestinal continuity was performed. Continuity was reconstructed by a retrosternal, isoperistaltic interposition of the right colon with an end-to-end esophagocolonic anastomosis, an end-to-end cologastric anastomosis and an end-to-end anastomosis of the terminal ileum and colon transversum.

In the following course the abdominal wall was reconstructed by a component separation technique according to Oscar Ramirez [4].

In the further course, our patient developed a collar fistula of the proximal anastomosis and a stenosis of the esophageal anastomosis of the colon interponat 20 to 25cm (measured from teeth) with a remaining lumen of 5mm, which was treated by placement of a covered esophageal stent. Multiple stent reimplantations followed, with periods without a stent to avoid pressure necrosis, with a good result. The stent could finally be removed approximately three years after the initial surgery.

**Figure 2:**
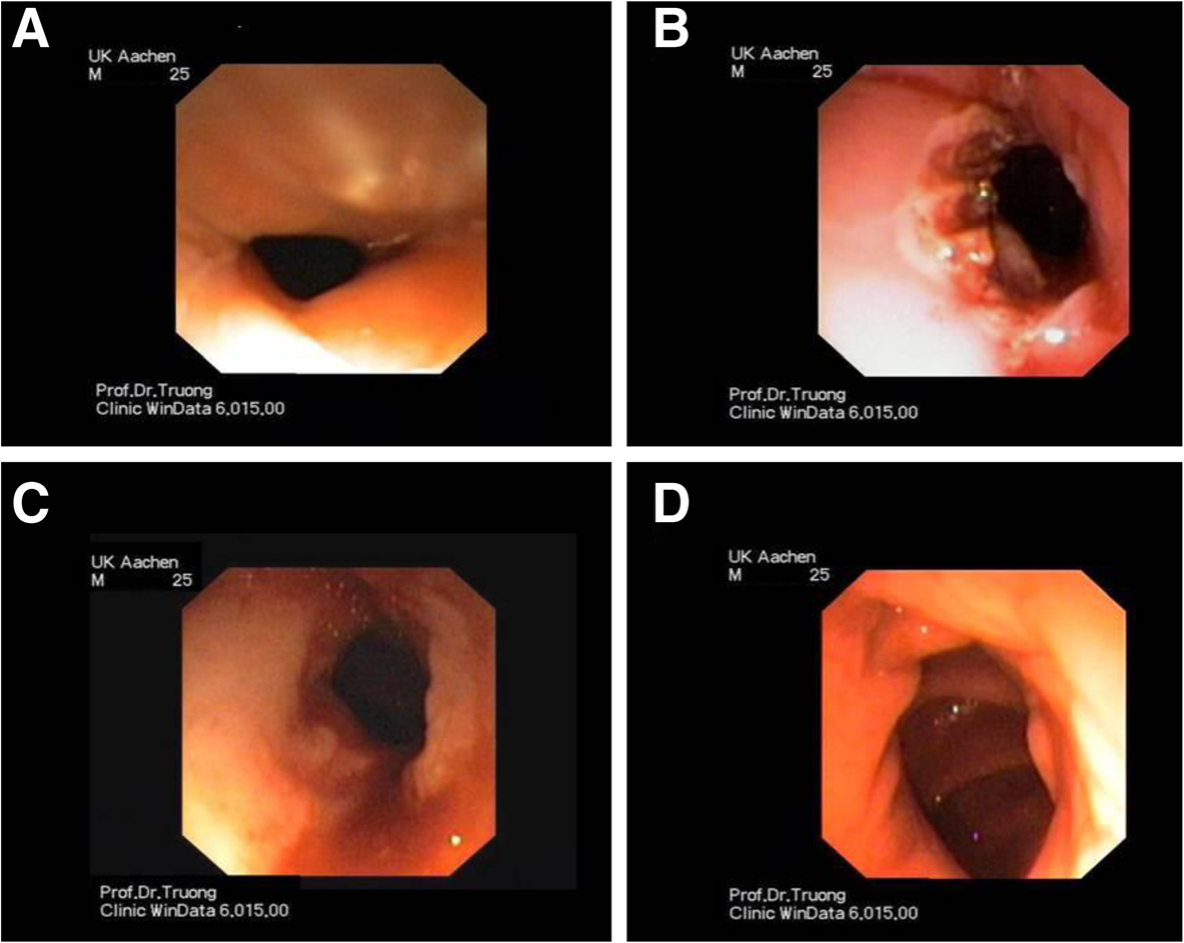
**Endoscopic illustration of a stenosis of the esophagocolonic anastomosis that was dilated endoluminally using the Savary-Gillard technique. A** Initial stenosis (7mm), **B** Result after dilatation and argon-plasma coagulation, **C** Follow-up showing scar tissue with re-stenosis, **D** End-result after recurrent dilatations (12mm).

Our patient has been followed up for more than six years in our outpatient clinic. Generally, he has almost completely recovered. Today, he is in an adequate nutritional and general condition. Due to the extensive course of the disease a consequent socioprofessional reintegration was required. Unfortunately, since the removal of the esophageal stent repetitive anastomotic stenoses occurred (Figure 2), which were treated by endoscopic dilatations using the Savary-Gilliard technique.

## Discussion

We present the successful management of a case of fulminant IM with extremely aggressive progression. Fulminant infectious mononucleosis is known to develop in patients with X-linked lymphoproliferative syndrome (XLP) or patients with immunodeficiency. However, some patients even develop fatal IM without any obvious preexisting immune abnormality - as presented in this case [5].

To the best of our knowledge, the combined occurrence of diffuse mucosal bleeding, splenomegaly with subsequent rupture, esophageal and gastric ulceration with spontaneous perforation, as well as hepatitis with hepatic failure altogether caused by an EBV infection in an otherwise healthy man has not been reported until today. Genetic testing to clarify if the patient suffered from XLP was not performed on our patient.

On primary examination, 50 to 60% of patients with EBV infection have splenomegaly, with splenic rupture occurring in 0.5% with a lethality of 30% [6-8]. In these patients emergency splenectomy is often necessary. Hepatomegaly is present in about 10% of adult patients with primary EBV infection. Mild liver function abnormalities occur in 80% of the patients and mostly during the second week of illness. Mainly, they are self-limiting and resolve three weeks after the onset of the disease [6]. Liver enzymes (aspartate aminotransferase (AST) and alanine aminotransferase (ALT)) might be increased up to fivefold values. The level of bilirubin is mostly mildly increased, which might be due to hemolytic anemia or hepatitis. Only 2% of patients are icteric [6]. Severe liver injury is rare and can lead to death in acute EBV infection [9,10].

Other cases of renal failure due to EBV infection have been described [11]. However, whether the renal failure observed in our case might have been partially due to aprotinin administration or EBV infection alone remains unclear. Aprotinin has been used in the treatment of adult patients with major blood loss during or following surgery in the past. It was shown to cause renal failure and therefore was voluntarily withdrawn by the manufacturer in 2008.

The development of a deep esophageal and gastric ulcer through EBV infection has only been described in association with AIDS or other immunodeficiencies [12,13]. The observed mucosal bleeding was most likely due to impaired coagulation caused by hepatic failure and thrombocytopenia caused by hypersplenism, as well as local inflammation of the mucosal tissue. The pronounced inflammation of the mucosa even led to a deep ulcer of the esophagus with subsequent spontaneous perforation and arterial hemorrhage making emergency surgical treatment necessary. In the further course, impaired wound healing led to necrosis of the sutured esophagus necessitating resection. This extremely complicated the course of the disease for the patient.

Antimicrobial therapy with antibiotics was given only on basis of clinical symptoms, because bacterial or superinfection was suspected. However, in none of the collected probes was a bacterial or fungal germ found. Whereas a benefit of antiviral therapy has been described in some cases [5], its overall benefit remains unclear. Controlled trials on treatment with aciclovir in patients with EBV have shown that treatment neither reduces the severity of clinical symptoms nor their duration [13,14]. Therefore no antiviral therapy was administered in our case.

## Conclusions

Although primary EBV infection is rarely fatal, fulminant infection may occur - in this case leading to hepatic failure, splenic rupture and esophageal necrosis that required surgical interventions. Our report shows, that in rare situations infectious mononucleosis is not only a medical condition but can also lead to severe complications including the need for surgical intervention. This case is an example of successful interdisciplinary management of complicated fulminant infectious mononucleosis in an otherwise healthy 18-year-old Caucasian man.

## Consent

Written informed consent was obtained from the patient for publication of this case report and any accompanying images. A copy of the written consent is available for review by the Editor-in-Chief of this journal.

## Abbreviations

AIDS: Acquired immune deficiency syndrome; ALT: Alanine aminotransferase; AST: Aspartate aminotransferase; AT III: Antithrombin III; CMV: Cytomegalovirus; CT: Computed tomography; EBV: Epstein-Barr virus; HAV: Hepatitis A virus; HBV: Hepatitis B virus; HCV: Hepatitis C virus; HIV: Human immune deficiency virus; ICU: Intensive care unit; IM: Infectious mononucleosis; MODS: Multiorgan dysfunction syndrome; PPSB: Prothrombin complex; PCHE: Plasma cholinesterase; PCR: Polymerase chain reaction; PT: Prothrombin time; XLP: X-linked lymphoproliferative syndrome.

## Competing interests

The authors declare that they have no competing interests.

## Authors’ contributions

DB and MB collected and interpreted the data and were major contributors in writing and revising the manuscript. KTvT, SH, DSS and KJ were major contributors in writing and revising the manuscript. NG performed the histological investigations and revised the manuscript. ST performed the endoscopic procedures and critically revised the manuscript. UPN was involved in interpretation of the data and critically revised the manuscript. All authors read and approved the final manuscript.
